# Genetic Diversity and Population Structure of Wild *Macrobrachium nipponense* in the Pearl River Basin: A Treasury of Germplasm for Aquaculture and Conservation

**DOI:** 10.3390/ani16182869

**Published:** 2026-09-11

**Authors:** Hao Dong, Xiaofan Fang, Yuefan Zhang, Shubo Jin, Wenyi Zhang, Yiwei Xiong, Hui Qiao, Sufei Jiang

**Affiliations:** 1 Key Laboratory of Mariculture & Stock Enhancement in North China’s Sea, Ministry of Agriculture and Rural Affairs, Dalian Ocean University, Dalian 116023, China; qwe1992208@163.com (H.D.); m15304476580@163.com (X.F.); 13377712674@163.com (Y.Z.); jinsb@ffrc.cn (S.J.); qiaoh@ffrc.cn (H.Q.); 2 Key Laboratory of Freshwater Fisheries and Germplasm Resources Utilization, Ministry of Agriculture and Rural Affairs, Freshwater Fisheries Research Center, Chinese Academy of Fishery Sciences, Wuxi 214081, China; zhangwy@ffrc.cn (W.Z.); xiongyw@ffrc.cn (Y.X.); 3 Wuxi Fisheries College, Nanjing Agricultural University, Wuxi 214081, China

**Keywords:** genetic diversity, population structure, *Macrobrachium nipponense*, D-loop, Pearl River basin

## Abstract

This study evaluated the genetic diversity and population structure of wild *M. nipponense* populations in the Pearl River Basin based on mitochondrial DNA (mtDNA) D-loop sequences. A total of 14 wild populations were collected from the West River, North River, and Pearl River Delta, and genetic diversity and population differentiation were assessed using haplotype analysis, analysis of molecular variance (AMOVA), pairwise *F*_ST_, and principal component analysis (PCA). A total of 351 variable sites were identified. The overall haplotype diversity (*h*) and nucleotide diversity (*π*) were 0.947 and 0.03665, respectively, indicating high genetic diversity across the Pearl River Basin. Significant genetic differentiation was detected among populations (*F*_ST_ = 0.3785, *p*-value < 0.001), with 37.85% of genetic variation occurring among populations. PCA revealed distinct genetic relationships among populations, supporting the presence of population genetic structure within the Pearl River Basin. Populations from Tian’e County, Xilin County, Dahua Yao Autonomous County, and other upper tributaries of the West River exhibited distinct genetic characteristics compared with populations from Zhaoqing City, Guiping City, Guangzhou City, and the middle and lower reaches of the West River and the Pearl River estuary. Among the 14 populations, those from Zhaoqing City, Guiping City, and Guangzhou City exhibited relatively high genetic diversity and distinctive genetic characteristics. These findings suggest that the complex hydrological structure and historical connectivity of the Pearl River Basin have contributed to the observed population genetic structure. The genetically diverse populations identified in this study represent valuable wild germplasm resources for the conservation and selective breeding of *M. nipponense*.

## Simple Summary

The oriental river prawn (*Macrobrachium nipponense*) is an important freshwater aquaculture species in China. However, the genetic characteristics of wild populations distributed throughout the Pearl River Basin remain poorly understood. In this study, wild *M. nipponense* populations were collected from 14 locations across the Pearl River Basin, and mitochondrial DNA sequences were analyzed to investigate genetic diversity and differentiation among populations. The results showed that wild populations in the Pearl River Basin harbor high levels of genetic variation and exhibit clear genetic differentiation among populations from different geographic regions. Several populations showed distinctive genetic characteristics and may represent valuable genetic resources for conservation and breeding. This study provides important information for the conservation and sustainable utilization of wild *M. nipponense* populations and may contribute to the development of improved aquaculture strains in the future.

## 1. Introduction

*M. nipponense*, commonly known as the oriental river prawn, is an economically and ecologically important freshwater crustacean distributed throughout China, Japan, and Southeast Asia [1,2]. In China, it is widely distributed in lakes, rivers, and reservoirs. Owing to its good meat quality, high nutritional value, high environmental adaptability, rapid growth, and high reproductive capacity, *M. nipponense* has become one of the major freshwater aquaculture species in China and is widely accepted by both farmers and consumers [3]. Large-scale culture of *M. nipponense* began in China in the mid-1960s and expanded rapidly during the 1990s. Early production was mainly concentrated in the middle and lower reaches of the Yangtze River, particularly in Jiangsu, Anhui, Zhejiang, and Jiangxi provinces. As market demand and prices increased, the rapid expansion of the industry also exposed several problems, including inconsistent broodstock sources, inadequate germplasm management, and the widespread use of commercial shrimp as breeding parents [4]. These factors contributed to the genetic deterioration of cultured stocks, as evidenced by slower growth, smaller body size, reduced disease resistance, and earlier sexual maturation. Consequently, aquaculture productivity gradually declined, and the gap between market supply and demand continued to widen, limiting the sustainable development of the *M. nipponense* industry [5,6]. To improve cultured germplasm and meet the growing demand for aquaculture production, our research group has conducted selective breeding of *M. nipponense* for more than 20 years. During this period, several improved varieties, with growth performance as the primary breeding objective, have been developed, including “Taihu No. 1”, “Taihu No. 2”, and “Taihu No. 3”. These varieties have helped improve the availability of high-quality germplasm for aquaculture. However, they may still not fully meet the production requirements of different farming regions across China.

The identification and utilization of genetically diverse wild germplasm as parental stocks are essential for maintaining the long-term sustainability of cultured *M. nipponense* and supporting the sustainable development of the aquaculture industry. In recent years, molecular markers have been widely used to investigate the genetic diversity and population structure of wild *M. nipponense* populations in China. Previous studies have focused on major distribution areas, including the Yangtze River Basin, the Yellow River Basin, and major lakes and rivers in Jiangsu and Zhejiang Provinces, which are important aquaculture regions in China, and have identified several wild populations with potential breeding value [7,8,9]. Based on these studies, our research group analyzed the genetic diversity and population structure of 22 wild *M. nipponense* populations collected from major rivers and lakes across China using mitochondrial DNA D-loop sequences. The results revealed clear differences in genetic diversity and population differentiation among geographic populations. In particular, populations in southern China, including those from the Pearl River, Min River, and Nandu River, exhibited relatively high genetic diversity and distinctive genetic characteristics [7].

The Pearl River is the largest river system in southern China and consists of the West River, North River, East River, and the Pearl River Delta river network. It flows through eight provincial-level administrative regions, including Yunnan, Guizhou, Guangxi, Guangdong, Hunan, Jiangxi, Fujian, and Hainan, and is an important region for hydropower development in China. The Pearl River Basin supports rich aquatic biodiversity and represents an important reservoir of aquatic genetic resources in southern China, as well as a major freshwater fishery production area. However, the genetic diversity and population structure of wild *M. nipponense* populations in the Pearl River Basin have received relatively little attention [7,9]. The mitochondrial DNA (mtDNA) control region (D-loop) is one of the most variable regions of the mitochondrial genome and evolves more rapidly than many other mtDNA regions. Owing to its high sequence polymorphism and ability to detect genetic variation among closely related populations, the D-loop has been widely used in population genetic studies of crustaceans and other aquatic animals, particularly for investigating genetic differentiation, phylogeographic patterns, and population relationships [10,11,12]. Previous studies have evaluated the genetic diversity of *M. nipponense* using various molecular markers, including mitochondrial *COI* gene sequences [1], microsatellite markers [13], mitochondrial *COI* and *16S rRNA* gene sequences [13], and amplified fragment length polymorphism (AFLP) markers [14].

Given the complex drainage network of the Pearl River Basin, which consists of multiple tributaries and estuarine systems, we hypothesized that differences in hydrological connectivity and geographic separation may have contributed to genetic differentiation among wild *M. nipponense* populations. To test this hypothesis, mtDNA D-loop sequences were used to investigate the genetic diversity and population genetic structure of 14 wild *M. nipponense* populations collected from the upper, middle, and lower reaches of the Pearl River Basin. The findings provide new insights into the genetic characteristics of wild *M. nipponense* populations and provide valuable information for germplasm conservation, sustainable resource utilization, and future selective breeding programs.

## 2. Materials and Methods

### 2.1. Ethics Statement

No endangered or protected species were involved in this study. All experimental procedures were reviewed and approved by the Animal Care and Use Ethics Committee of the Freshwater Fisheries Research Center, Chinese Academy of Fishery Sciences (Wuxi, China) (Approval No. 20250901003).

### 2.2. Sample Collection

**Figure 1:**
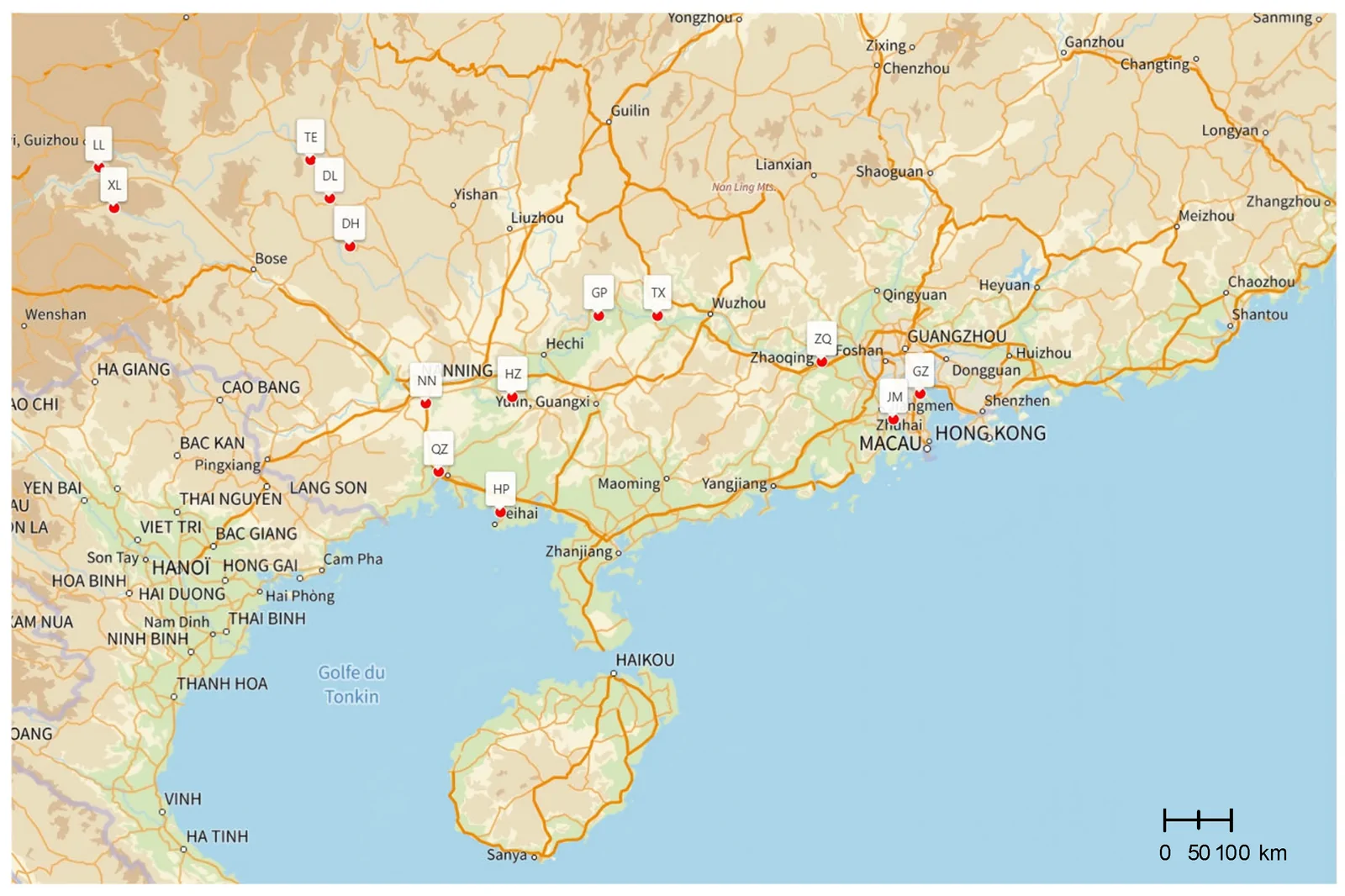
Sampling sites, coordinates, and sample sizes of *M. nipponense* in the Pearl River Basin, China.

Wild *M. nipponense* individuals were collected using prawn cages at sampling sites in Guangdong and Guangxi Provinces within the Pearl River Basin, as shown in Figure 1. The collected specimens were identified as *M. nipponense* based on the taxonomic descriptions provided in Fauna Sinica, Invertebrates, Volume 44: Subphylum Crustacea, Decapoda, Long-brachioidea [15]. Basic information on all sampling sites is summarized in Table 1. A portion of abdominal muscle tissue from each individual was excised and preserved in 95% ethanol for subsequent DNA extraction.

### 2.3. DNA Preparation, PCR Amplification, and Sequencing

Approximately 100 wild individuals were initially collected from each population. For DNA extraction and sequencing, approximately 35 individuals per population were randomly selected. Approximately 50 mg of abdominal muscle tissue was collected from each individual, and genomic DNA was extracted using the phenol–chloroform method [16]. DNA concentration and purity were measured using a microvolume spectrophotometer (Thermo Fisher Scientific, Waltham, MA, USA) and further verified by 1.2% agarose gel electrophoresis. DNA samples were diluted to approximately 50 ng/μL and stored at −20 °C until further analysis. The mitochondrial DNA (mtDNA) D-loop primers used in this study were previously designed by our research group [17]. The primer sequences were as follows: forward, 5′-TTTACTCCCAGTCTAACC-3′; reverse, 5′-TTCATTATTCGCCCTATC-3′. The PCR reaction mixture and thermal cycling conditions were the same as those described by Jiang et al. [17]. PCR products were examined by 1.5% agarose gel electrophoresis, and products showing a single, clear band of the expected size were subjected to bidirectional Sanger sequencing. Sequencing was performed by Sangon Biotech Co., Ltd. (Shanghai, China). The obtained sequences were assembled and manually inspected to minimize potential base-calling errors. Sequences with less than 90% identity or coverage, or with excessive gaps or missing data, were excluded from further analyses. For populations with fewer than 30 qualified sequences, additional samples were sequenced until at least 30 high-quality sequences were obtained. Finally, 30 sequences from each of the 14 populations, yielding a total of 420 sequences, were retained for subsequent analyses.

### 2.4. Data Analysis

To further confirm species identity and sequence quality and to minimize the potential influence of nuclear mitochondrial pseudogenes (NUMTs), all obtained D-loop sequences were compared with reference sequences available in the NCBI database using the Basic Local Alignment Search Tool (BLAST; https://blast.ncbi.nlm.nih.gov/, accessed on 24 October 2025) before subsequent population genetic analyses. Sequence alignment and sequence length determination were performed using BioEdit 7.0.9 [18]. Nucleotide diversity (*π*) and haplotype diversity (*h*) were calculated using DnaSP 6.0. Analysis of molecular variance (AMOVA) was conducted using ARLEQUIN 3.5 with 1000 permutations [19]. Genetic distances among populations were estimated in MEGA 7 using the Kimura two-parameter (K2P) model [20]. Pairwise genetic differentiation (*F*_ST_) was calculated in ARLEQUIN 3.5 with 10,000 permutations, and *p*-values were adjusted using the Benjamini–Hochberg false discovery rate (FDR) procedure [21]. A haplotype network was constructed using the median-joining algorithm implemented in POPART 1.7 [22]. A neighbor-joining phylogenetic tree was reconstructed in MEGA 7 based on genetic distances [23]. Principal component analysis (PCA) was performed using the adegenet package in R (R version 4.3.1) based only on variable polymorphic sites of the mtDNA D-loop sequences. PCA was used as an exploratory visualization tool to display genetic relationships among populations rather than as an independent statistical test of population structure (http://cran.r-project.org/mirrors.html, accessed on 24 October 2025).

## 3. Results

### 3.1. D-Loop Sequences Analysis

A total of approximately 490 wild *M. nipponense* individuals from 14 geographic populations across the Pearl River Basin were initially selected for DNA extraction and sequencing. After sequence quality control and additional sequencing where necessary, 420 high-quality D-loop sequences, each 1058 bp in length, were obtained. Thirty sequences from each population were retained for subsequent analyses (NCBI GenBank accession No. SUB16359777). Across all populations, 351 polymorphic sites were identified, although their numbers varied considerably among populations (Table 2). Five populations contained 150 or more polymorphic sites, with the GP and ZQ populations showing the highest numbers (216 and 217, respectively). Another five populations contained 50–150 polymorphic sites, whereas the remaining four populations contained fewer than 50 polymorphic sites. Among these, the TE and HP populations had the lowest numbers, with 22 and 31 polymorphic sites, respectively.

A total of 151 haplotypes were identified across the 14 geographic populations in the Pearl River Basin, and their distribution is summarized in Table 2. The ZQ and GZ populations each contained 30 haplotypes, the highest number among all populations, whereas the TX and XL populations contained only 7 and 9 haplotypes, respectively. As shown in Supplementary Table S1, Hap1, Hap2, Hap4, and Hap83 were the most widely distributed haplotypes, occurring in 7, 11, 9, and 8 populations, respectively. Hap6, Hap7, and Hap23 were each detected in five populations; Hap3, Hap5, and Hap25 in four populations; Hap9 and Hap11 in three populations; and Hap13, Hap15, Hap30, Hap56, Hap57, Hap66, and Hap101 in two populations. All other haplotypes were unique to a single population. The distribution of private haplotypes also varied among populations. The ZQ and GZ populations each possessed 30 private haplotypes, whereas no private haplotypes were detected in the DH, LL, or TE populations.

### 3.2. Population Genetic Diversity Analysis

The genetic diversity indices of the 14 *M. nipponense* populations are summarized in Table 2. Across all populations, the overall haplotype diversity (h) was 0.947, with values ranging from 0.678 to 1.000. Five populations showed h ≥ 0.900, including ZQ and GZ (1.000), followed by GP (0.952), QZ (0.936), and DL (0.917). In contrast, the HP population had the lowest haplotype diversity (0.678). The overall nucleotide diversity (π) was 0.03665, ranging from 0.00423 to 0.07177 among populations. The highest values were observed in GP (0.07177), followed by ZQ (0.06067), JM (0.03839), and QZ (0.03062). By comparison, TE, HP, and DH showed relatively low nucleotide diversity, with π values of 0.00423, 0.00446, and 0.00600, respectively. Comparison of the two diversity indices revealed marked differences among populations. The ZQ and GP populations were characterized by both high haplotype diversity and high nucleotide diversity, whereas the HP population exhibited relatively low values for both indices.

### 3.3. Population Genetic Variation Analysis

Analysis of molecular variance (AMOVA) was conducted to quantify the distribution of genetic variation within and among the 14 *M. nipponense* populations (Table 3). Genetic variation was significant both among and within populations (*p*-value < 0.01). Variation among populations accounted for 37.85% of the total genetic variation, whereas 62.15% was distributed within populations. The overall *F*_ST_ value was 0.37853, indicating substantial genetic differentiation among the 14 populations. According to commonly used criteria for interpreting *F*_ST_ values, this result indicates a high level of genetic differentiation among populations (*F*_ST_ > 0.25).

Pairwise *F*_ST_ values among the 14 *M. nipponense* populations are presented in Table 4. The *F*_ST_ values ranged from 0.000 between TE and DH to 0.711 between TE and GZ, indicating substantial variation in the degree of genetic differentiation among population pairs. Only 11 pairwise comparisons showed non-significant genetic differentiation (*p*-value > 0.05), including LL–DH, DH–DL, NN–HZ, HZ–JM, JM–NN, TX–HZ, TX–NN, TX–JM, QZ–DH, TE–DH, and TE–NN. All remaining pairwise comparisons showed significant genetic differentiation (*p*-value < 0.01). Among the downstream populations, HP exhibited relatively strong genetic differentiation from GZ, with an *F*_ST_ value greater than 0.5, whereas relatively lower differentiation was observed between HP and upstream populations such as TE, with *F*_ST_ values below 0.5. A total of 39 pairwise comparisons had *F*_ST_ values greater than 0.25. Notably, GP and ZQ showed *F*_ST_ values greater than 0.25 in comparisons with nearly all other populations, indicating pronounced genetic differentiation from most other populations.

### 3.4. Phylogeny and Population Genetic Structure

**Figure 2:**
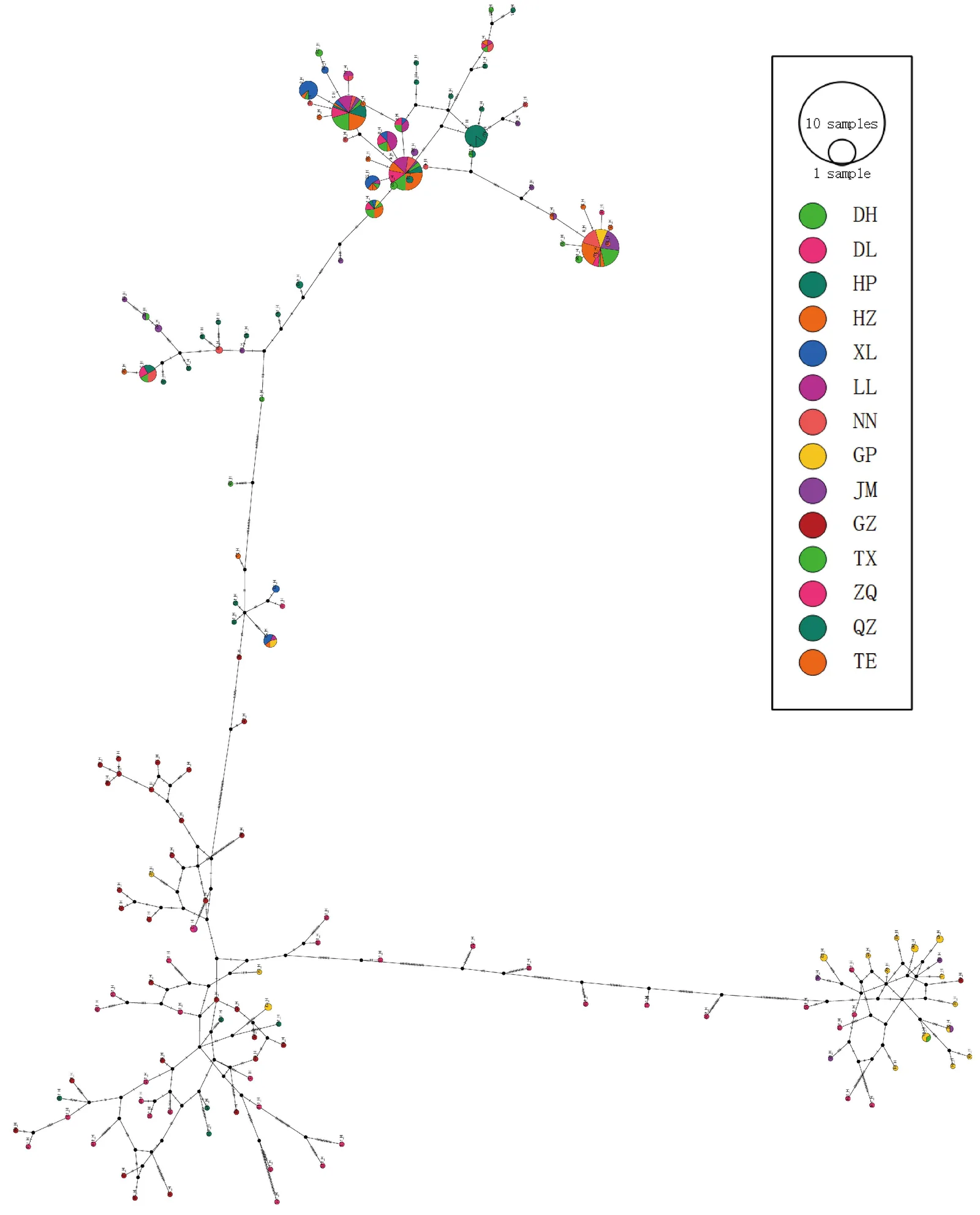
Median-joining network of mitochondrial D-loop haplotypes of *M. nipponense* populations in the Pearl River Basin. Each circle represents a haplotype, and the circle size is proportional to the number of individuals carrying that haplotype. Different colors indicate the corresponding sampling populations. The connecting lines represent mutational steps between haplotypes, and black circles indicate hypothetical missing intermediate haplotypes.

A haplotype network based on the 151 identified haplotypes is shown in Figure 2 and Supplementary Table S1. The network revealed clear differences in haplotype composition among populations. The XL, LL, and TE populations shared several common haplotypes, including Hap_2, Hap_4, and Hap_6, suggesting relatively close genetic relationships among these populations. In contrast, the HP population shared only a few haplotypes with other populations, indicating a relatively distinct haplotype composition. Although the GP population shared several haplotypes, including Hap_8, Hap_57, and Hap_66, with the JM and TX populations, it also contained a large number of private haplotypes, indicating substantial genetic diversity and a distinctive haplotype composition. Similarly, the GZ and ZQ populations exhibited high haplotype diversity but shared very few haplotypes with each other, suggesting marked differences in their genetic composition.

**Figure 3:**
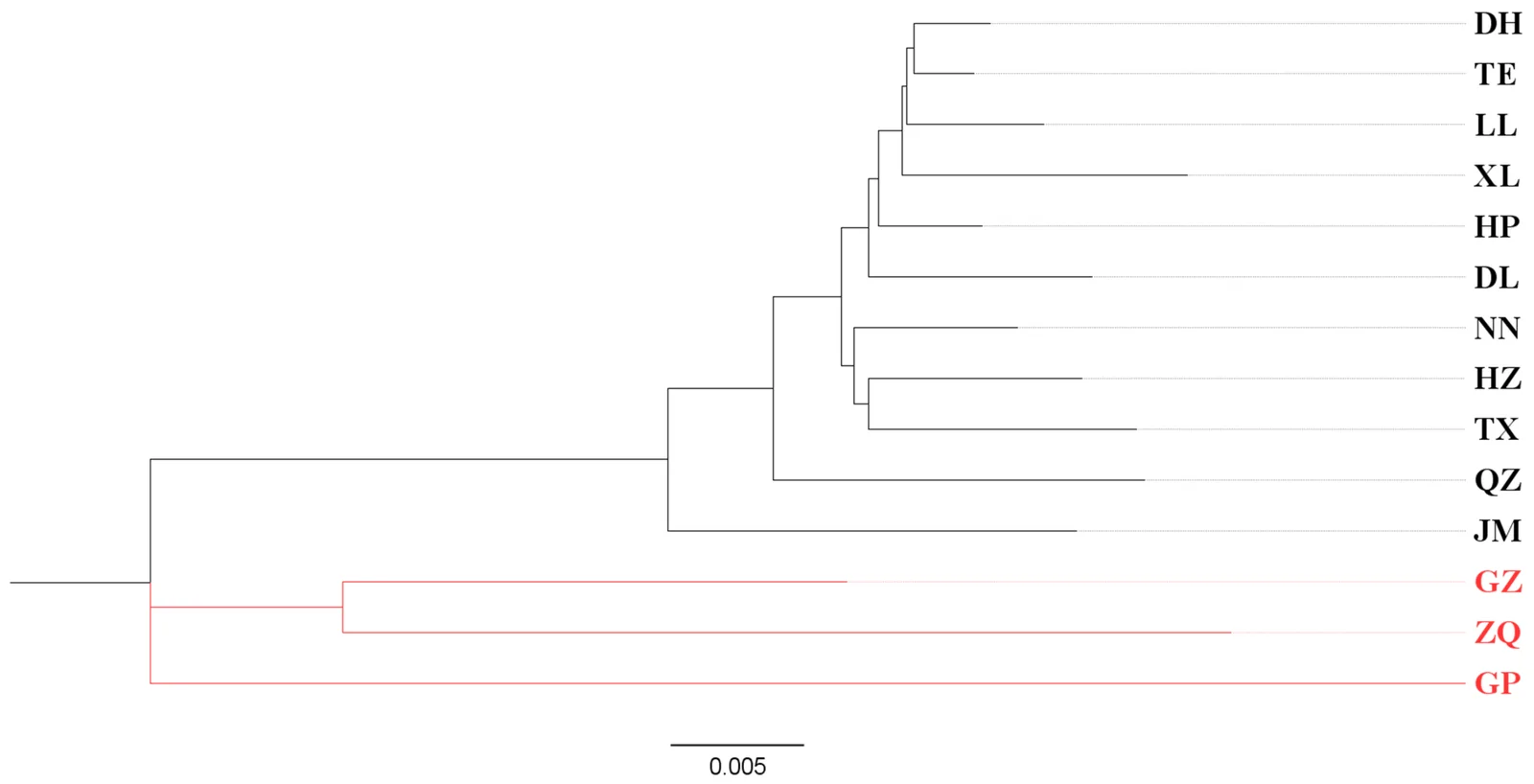
Phylogenetic tree based on genetic distances of 14 *M. nipponense* sampling sites. The red branches highlight the GZ, ZQ, and GP populations.

Pairwise genetic distances among the 14 *M. nipponense* populations are presented in Table 5. Genetic distances ranged from 0.00449 to 0.08301. The greatest genetic distance was detected between the GP and GZ populations (0.08301), followed by the GP–XL (0.08179), GP–LL (0.08050), and GP–DL (0.08026) population pairs. In contrast, the smallest genetic distance (0.00449) was found between the DH and TE populations. A phylogenetic tree was subsequently constructed using the pairwise genetic distance matrix (Figure 3). The resulting topology separated the 14 populations into two major clusters. Group I included populations from the Hongshui River, the Tuoniang River, and several tributaries of the West River (e.g., TE, XL, and DH), whereas Group II comprised populations from the middle and lower reaches of the West River together with those from the estuarine region (ZQ, GP, and GZ).

**Figure 4:**
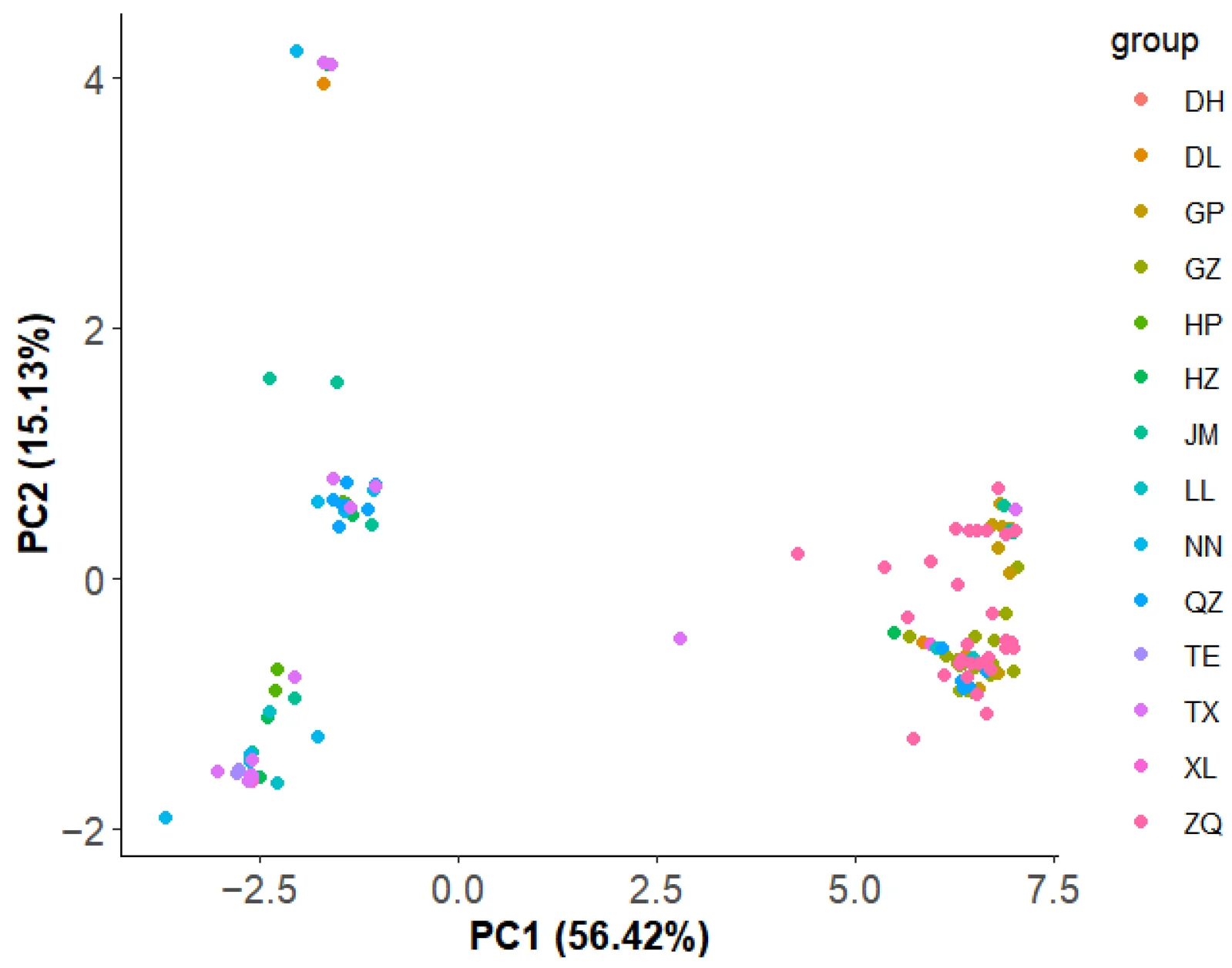
The principal components analysis (PCA) of 14 *M. nipponense* sites showing two main clusters. Each color represents a specific site.

Principal component analysis (PCA) was conducted to visualize the genetic relationships among the 14 *M. nipponense* populations (Figure 4). The PCA revealed clustering patterns that were generally consistent with those observed in the phylogenetic analysis. The populations were broadly separated into two major groups. Group I comprised populations from the Hongshui River, the Tuoniang River, and several tributaries of the West River, including TE, XL, and DH, whereas Group II comprised populations from the middle and lower reaches of the West River and the Pearl River estuary, including ZQ, GP, and GZ. Although TE was assigned to Group I, it occupied a relatively distinct position within this group, suggesting some degree of genetic differentiation from the other upstream populations.

## 4. Discussion

Unlike the Yangtze and Yellow rivers, which are characterized by relatively simple main-channel systems, the Pearl River Basin has a complex multi-trunk drainage network [24]. It comprises the West, North, and East rivers, together with the distributary channels of the Pearl River Delta. Multiple tributaries converge from different directions before entering the South China Sea through several estuaries. Among these rivers, the West River is the largest and forms the principal drainage system of the basin. The sampling sites in the present study covered representative locations from the upper reaches to the estuary of the West River, including the Nanpan River (LL), Hongshui River (DL, DH, and TE), Tuoniang River (XL), Yong River (NN), Yu River (HZ), and Xun River (GP). Additional populations were sampled from the West River mainstem and its major tributaries, including the Gui River (ZQ), He River (JM), and Beiliu River (GZ), as well as the independently flowing Nanliu River (QZ) and Qin River (HP). This broad geographic coverage allowed genetic patterns to be considered in the context of the complex drainage configuration of the Pearl River Basin. In particular, the Xun River, where the Qian and Yu rivers converge, receives water from both the Hongshui and Yu rivers, placing the GP population at an important hydrological junction between the upper and lower reaches of the basin. Such differences in drainage connectivity may influence opportunities for dispersal and gene flow among *M. nipponense* populations and may therefore contribute to the genetic differentiation observed in the present study.

Genetic diversity reflects the extent of genetic variation within and among populations and provides the evolutionary potential necessary for populations to respond to environmental change, predation, parasitism, and pathogen pressure [25,26,27]. In the present study, mitochondrial D-loop sequences revealed substantial genetic variation among the 14 *M. nipponense* populations from the Pearl River Basin. Haplotype diversity (*h*) and nucleotide diversity (*π*) provide complementary information for evaluating genetic diversity and the evolutionary history of natural populations [28]. The nucleotide diversity observed in the present study fell within the range previously reported for wild *M. nipponense* populations based on mitochondrial D-loop sequences, which ranged from 0.0011 to 0.0607 among populations [9]. Previous studies have also reported considerable variation in genetic diversity among wild *M. nipponense* populations from different geographic regions, including saline–alkaline regions of China [29] and multiple river basins across the country [7]. Consistent with these findings, Zhang et al. [9] reported an average haplotype diversity of 0.844 across eight *M. nipponense* populations, whereas Jiang et al. [7] reported a maximum nucleotide diversity of 0.037168 among 22 populations. Comparisons with several freshwater fishes [30,31,32,33,34,35] provide additional context for evaluating genetic variation in aquatic organisms; however, such cross-taxon comparisons should be interpreted cautiously because differences in evolutionary history, life-history traits, ecological characteristics, and molecular markers may influence estimates of genetic diversity. Overall, these comparisons suggest that the populations examined in the present study maintain relatively high levels of genetic diversity. The substantial genetic variation observed across the Pearl River Basin may be associated with its complex drainage network, broad geographic coverage, and historical connectivity among tributary systems, which may have contributed to the retention of diverse maternal lineages.

Analysis of molecular variance (AMOVA) was performed to evaluate the distribution of genetic variation within and among *M. nipponense* populations in the Pearl River Basin. The results showed that 37.85% of the total genetic variation occurred among populations (*F*_ST_ = 0.3785, *p*-value), whereas 62.15% occurred within populations, indicating that within-population variation represented the major component of total genetic variation despite substantial differentiation among populations. Previous studies of wild *M. nipponense* have similarly shown that a considerable proportion of genetic variation is retained within populations, although substantial genetic differentiation may also occur among geographically separated populations [1,7,30]. The predominance of within-population variation observed in the present study is consistent with the relatively high genetic diversity detected across the Pearl River Basin, whereas the significant among-population component, together with the pairwise *F*_ST_ and PCA results, supports the presence of a pronounced population genetic structure. Overall, these findings suggest that genetic variation in the Pearl River Basin is maintained at both within- and among-population levels, with the observed population structure potentially influenced by complex hydrological connectivity and historical processes.

The genetic distance matrix and PCA further supported genetic differentiation among *M. nipponense* populations in the Pearl River Basin. PCA revealed patterns of genetic relationships among populations and provided complementary evidence for the population structure inferred from mitochondrial DNA-based analyses. Significant pairwise *F*_ST_ values among several populations, together with the AMOVA results, further supported the presence of substantial genetic differentiation within the basin. Among the 14 populations, GP exhibited particularly distinctive genetic characteristics, including the highest nucleotide diversity and relatively pronounced genetic differentiation from several other populations. GP was also involved in several of the largest pairwise genetic distances observed in this study, further supporting its distinctive genetic composition. Notably, GP is located in an important geographical transition zone within the Pearl River Basin, where the complex hydrological setting may have contributed to its observed genetic characteristics. The combination of high genetic diversity, pronounced genetic differentiation, and its geographical location suggests that GP may represent a particularly valuable wild germplasm resource. ZQ and GZ also exhibited relatively high genetic diversity and distinctive genetic characteristics, suggesting that they may have retained unique genetic variation. More broadly, the genetic patterns observed across the Pearl River Basin may be associated with its complex hydrological configuration and historical connectivity. Although the sampling sites encompassed upstream, middle, and downstream regions, the observed genetic differentiation did not follow a simple upstream–downstream pattern or a strict isolation-by-distance pattern. Instead, drainage configuration, tributary isolation, historical hydrological connectivity, and dispersal opportunities may have jointly influenced the population structure of *M. nipponense*. Differences in local hydrological conditions and connectivity among sub-basins may have restricted gene flow among some populations, suggesting that geographic distance alone cannot fully explain the observed genetic relationships within this complex river system. Collectively, these findings highlight the importance of considering both genetic diversity and genetic differentiation in the management and conservation of wild *M. nipponense* resources, particularly when identifying populations with unique genetic characteristics as potential priorities for germplasm conservation and future genetic improvement.

Cascade dams and hydropower stations are widely distributed across the upper Hongshui River and adjacent reaches and may act as barriers to gene flow in freshwater organisms. However, the populations sampled from these upstream areas exhibited relatively low levels of genetic differentiation and close genetic relationships, suggesting that the existing dam structures have not yet resulted in detectable genetic subdivision among upstream *M. nipponense* populations based on the mitochondrial D-loop marker. Nevertheless, the long-term effects of river fragmentation on population connectivity remain unclear and warrant further investigation. It should also be noted that the mitochondrial D-loop marker reflects only maternal genetic variation and may not fully capture genome-wide patterns of population structure and connectivity. Future studies integrating nuclear markers, including SNPs and other genome-wide approaches, with broader geographic sampling and environmental data could provide a more comprehensive understanding of gene flow, demographic history, and the factors shaping genetic differentiation. Such integrated approaches would further support the development of effective conservation strategies and the sustainable utilization of wild *M. nipponense* genetic resources.

## 5. Conclusions

This study provides an assessment of the genetic diversity and population structure of wild *M. nipponense* populations in the Pearl River Basin based on mitochondrial D-loop sequences. The results revealed that the 14 populations maintained high genetic diversity and exhibited substantial genetic differentiation, with the observed genetic patterns likely influenced by the complex hydrological connectivity and historical processes of the river system. Among these populations, ZQ, GP, and GZ showed relatively high genetic diversity and distinct genetic characteristics, suggesting that they represent valuable wild germplasm resources within the Pearl River Basin. These findings provide valuable genetic information for the conservation and sustainable utilization of wild *M. nipponense* resources. Populations with high genetic diversity and unique genetic characteristics may provide valuable genetic resources for future selective breeding and genetic improvement programs. However, as the mitochondrial D-loop marker represents only maternal genetic variation, future studies integrating nuclear markers, genome-wide approaches, and environmental variables are needed to further clarify population connectivity and adaptive variation. Such approaches will improve our understanding of the mechanisms underlying genetic differentiation in *M. nipponense*.

## Figures and Tables

**Table 1:** Sample information of *M. nipponense*, geographical position (Lat-Long), sampling date (yyyy-mm) and sample size (*n*).

Abbreviated Name	Population Geographical Position	Lat-Long	Sampling Date	Sample Size
LL	Longlin County, Baise City, Guangxi Zhuang Autonomous Region	105.03° E, 24.86° N	11 July 2025	100
XL	Xilin County, Baise City, Guangxi Zhuang Autonomous Region	105.18° E, 24.48° N	12 July 2025	100
TE	Tian’e County, Hechi City, Guangxi Zhuang Autonomous Region	107.21° E, 24.93° N	11 July 2025	100
DL	Donglan County, Hechi City, Guangxi Zhuang Autonomous Region	107.4° E, 24.57° N	10 July 2025	100
DH	Dahua Yao Autonomous County, Hechi City, Guangxi Zhuang Autonomous Region	107.61° E, 24.12° N	10 July 2025	100
HZ	Hengzhou City, Guangxi Zhuang Autonomous Region	109.28° E, 22.69° N	14 November 2024	100
NN	Nanning City, Guangxi Zhuang Autonomous Region	108.39° E, 22.63° N	14 November 2024	100
QZ	Qinzhou City, Guangxi Zhuang Autonomous Region	108.52° E, 21.98° N	15 November 2024	100
HP	Beihai City, Guangxi Zhuang Autonomous Region	109.16° E, 21.59° N	15 November 2024	100
GP	Guiping City, Guangxi Zhuang Autonomous Region	110.16° E, 23.45° N	14 October 2023	100
TX	Teng County, Wuzhou City, Guangxi Zhuang Autonomous Region	110.76° E, 23.45° N	13 October 2023	100
ZQ	Zhaoqing City, Guangdong Province	112.47° E, 23.02° N	14 October 2023	100
JM	Jiangmen City, Guangdong Province	113.19° E, 22.47° N	14 October 2023	100
GZ	Guangzhou City, Guangdong Province	113.47° E, 22.72° N	15 October 2023	100

**Table 2:** Summary statistics for D-loop polymorphisms of *M. nipponense.*

Population	Number of Polymorphic Sizes	Haplotype	Haplotype Diversity (h)	Nucleotide Diversity (π)	Private Haplotypes
DH	37	11	0.848 ± 0.047	0.00600 ± 0.00137	0
DL	119	13	0.917 ± 0.026	0.01870 ± 0.00477	3
HP	31	11	0.678 ± 0.094	0.00446 ± 0.00092	8
HZ	93	15	0.807 ± 0.071	0.01599 ± 0.00402	7
XL	76	9	0.809 ± 0.058	0.01986 ± 0.00551	4
LL	101	10	0.869 ± 0.033	0.01085 ± 0.00463	0
NN	45	13	0.883 ± 0.042	0.01264 ± 0.00085	8
GP	216	19	0.952 ± 0.026	0.07177 ± 0.00551	18
JM	158	16	0.841 ± 0.065	0.03839 ± 0.01006	9
GZ	187	30	1.000 ± 0.009	0.02730 ± 0.00664	30
TX	172	15	0.862 ± 0.058	0.02078 ± 0.00704	10
ZQ	217	30	1.000 ± 0.009	0.06067 ± 0.00572	30
QZ	142	19	0.936 ± 0.032	0.03062 ± 0.00483	16
TE	22	7	0.736 ± 0.054	0.00423 ± 0.00107	0
Total	351	151	0.9472 ± 0.0054	0.03665 ± 0.00224	143

**Table 3:** Analysis of molecular variance (AMOVA) results for the *M. nipponense*.

Source of Variation	df	Sum of Squares	Variance Components	Percentage of Variation
Among populations	13	2856.940	6.94540 Va	37.85
With in populations	406	4629.500	11.40271 Vb	62.15
Total		419	7486.440	18.34811
Fixation Index	*F*_ST_ = 0.37853 (*p*-value < 0.01)			

**Table 4:** Matrix of pairwise *F*_ST_ values among 14 sampling sites of *M. nipponense.*

	DH	DL	HP	HZ	XL	LL	NN	GP	JM	GZ	TX	ZQ	QZ	TE
DH	0	0.0540	0.00 *	0.00 *	0.03604	0.3964	0.00	0.00 *	0.00 *	0.00 *	0.00 *	0.00 *	0.00 *	0.4774
DL	0.034	0	0.00 *	0.02703	0.00 *	0.16216	0.04505	0.00 *	0.00 *	0.00 *	0.00 *	0.00 *	0.07207	0.00 *
HP	0.313	0.132	0	0.00 *	0.00 *	0.00 *	0.00 *	0.00 *	0.00 *	0.00 *	0.00 *	0.00 *	0.00 *	0.00 *
HZ	0.264	0.094	0.273	0	0.00 *	0.00 *	0.18018	0.00 *	0.08108	0.00 *	0.85586	0.00 *	0.00 *	0.00 *
XL	0.084	0.047	0.247	0.193	0	0.03604	0.00 *	0.00 *	0.00 *	0.00 *	0.00 *	0.00 *	0.00 *	0.00 *
LL	0	0.017	0.242	0.222	0.044	0	0.00 *	0.00 *	0.00 *	0.00 *	0.00 *	0.00 *	0.00 *	0.00 *
NN	0.189	0.043	0.215	0.018	0.182	0.179	0	0.00 *	0.06306	0.00 *	0.41441	0.00 *	0.00 *	0.4504
GP	0.528	0.460	0.529	0.462	0.465	0.507	0.476	0	0.00 *	0.00 *	0.00 *	0.00 *	0.00 *	0.00 *
JM	0.207	0.101	0.204	0.047	0.180	0.191	0.054	0.297	0	0.00 *	0.26126	0.00 *	0.00 *	0.00 *
GZ	0.691	0.571	0.691	0.604	0.584	0.650	0.623	0.415	0.477	0	0.00 *	0.00 *	0.00 *	0.00 *
TX	0.196	0.067	0.204	0	0.172	0.175	0	0.427	0.012	0.577	0	0.00 *	0.00 *	0.00 *
ZQ	0.529	0.440	0.526	0.464	0.456	0.500	0.474	0.167	0.330	0.145	0.435	0	0.00 *	0.00 *
QZ	0.149	0.035	0.156	0.146	0.092	0.122	0.103	0.402	0.110	0.423	0.118	0.336	0	0.00
TE	0	0.064	0.387	0.289	0.106	0.002	0.235	0.539	0.224	0.711	0.216	0.543	0.188	0

Pairwise *F*_ST_ (below the diagonal); significance of corresponding *p*-values (above the diagonal) based on pairwise differences in the concatenated mtDNA sequences. *: *p* < 0.05 as per false discovery rate correction [22].

**Table 5:** Genetic distances of 14 sampling sites of *M. nipponense.*

	DH	DL	HP	HZ	XL	LL	NN	GP	JM	GZ	TX	ZQ	QZ	TE
DH	0													
DL	0.01151	0												
HP	0.00705	0.01206	0											
HZ	0.01391	0.01751	0.01319	0										
XL	0.01264	0.01805	0.01449	0.02021	0									
LL	0.00753	0.01340	0.00907	0.01580	0.01424	0								
NN	0.01128	0.01544	0.01081	0.01415	0.01860	0.01363	0							
GP	0.07991	0.08026	0.07854	0.07882	0.08179	0.08050	0.07889	0						
JM	0.02678	0.02990	0.02584	0.02726	0.03329	0.02879	0.02631	0.07601	0					
GZ	0.05327	0.05135	0.05108	0.05346	0.05413	0.05281	0.05316	0.08301	0.06155	0				
TX	0.01582	0.01966	0.01515	0.01720	0.02269	0.01788	0.01632	0.07835	0.02881	0.05592	0			
ZQ	0.07010	0.06901	0.06835	0.07054	0.07186	0.07015	0.06977	0.07834	0.07274	0.05138	0.07125	0		
QZ	0.01957	0.02307	0.01894	0.02503	0.02500	0.02128	0.02262	0.08169	0.03639	0.04777	0.02705	0.06654	0	
TE	0.00449	0.01084	0.00631	0.01303	0.01185	0.00658	0.01069	0.07967	0.02612	0.05340	0.01498	0.07022	0.01930	0

## Data Availability

The original contributions presented in the study are included in the article; further inquiries can be directed to the corresponding authors.

## Supplementary Materials

The following supporting information can be downloaded at: https://www.mdpi.com/article/10.3390/ani16182869/s1, Table S1: Haplotype frequencies across 14 *Macrobrachium nipponense* populations.

## References

[1] Wang D., Hu L., Zhang F.B., Zheng F.Q., Gong M.Y., Xiong F., Liu H.Y., Zhai D.D. (2024). Genetic structure of *Macrobrachium nipponense*, an important farmed freshwater shrimp in China, in the Three Gorges Reservoir. Aquat. Living Resour..

[2] Cai Y.X., Shokita S. (2006). Report on a collection of freshwater shrimps (Crustacea: Decapoda: Caridea) from the Philippines, with descriptions of four new species. Raffles Bull. Zool..

[3] Cui F., Yu Y.Y., Bao F.Y., Wang S., Xiao M.S. (2018). Genetic diversity analysis of the oriental river prawn (*Macrobrachium nipponense*) in Huaihe River. Mitochondrial DNA Part A.

[4] Fu H.T., Jiang S.F., Xiong Y.W. (2012). Current status and prospects of farming the giant river prawn (*Macrobrachium rosenbergii*) and the oriental river prawn (*Macrobrachium nipponense*) in China. Aquac. Res..

[5] Jin S.B., Bian C., Jiang S.F., Han K., Xiong Y.W., Zhang W.Y., Shi C.C., Qiao H., Gao Z.J., Li R.H. (2021). A chromosome-level genome assembly of the oriental river prawn, *Macrobrachium nipponense*. GigaScience.

[6] Fu H.T., Gong Y.S., Wu Y., Xu P., Wu C.J. (2004). Artificial interspecific hybridization between *Macrobrachium nipponense Macrobrachium hainanense* and their isozyme analysis. Aquaculture.

[7] Jiang S., Xiong Y., Zhang L., Zhang W., Zheng Y., Wang J., Jin S., Gong Y., Wu Y., Qiao H. (2023). Genetic diversity and population structure of wild *Macrobrachium nipponense* populations across China: Implication for population management. Mol. Biol. Rep..

[8] Jiang H.-C., Feng J.-B., Ding H.-Y., Wang G.-L., Li J.-L. (2012). Genetic Structure and Phylogeography of Natural *Macrobrachium nipponense* Populations in Huaihe River Based on Mitochondrial *COI* Sequence. Chin. J. Zool..

[9] Zhang W., Jiang S., Salumy K.R., Xuan Z., Xiong Y., Jin S., Gong Y., Wu Y., Qiao H., Fu H. (2022). Comparison of genetic diversity and population structure of eight *Macrobrachium nipponense* populations in China based on D-loop sequences. Aquac. Rep..

[10] Zhang Q., Sun C., Zhu Y., Xu N., Liu H. (2020). Genetic diversity and structure of the round-tailed paradise fish (*Macropodus ocellatus*): Implications for population management. Glob. Ecol. Conserv..

[11] Stobie C.S., Cunningham M.J., Oosthuizen C.J., Bloomer P. (2019). Finding stories in noise: Mitochondrial portraits from RAD data. Mol. Ecol. Resour..

[12] Gallagher J., Finarelli J.A., Jonasson J.P., Carlsson J. (2019). Mitochondrial D-loop DNA analyses of Norway lobster (*Nephrops norvegicus*) reveals genetic isolation between Atlantic and East Mediterranean populations. J. Mar. Biol. Assoc. U. K..

[13] Ma K.Y., Feng J.B., Li J.L. (2012). Genetic variation based on microsatellite analysis of the oriental river prawn, *Macrobrachium nipponense* from Qiandao Lake in China. Genet. Mol. Res..

[14] Ge J., Xu Z., Huang Y., Lu Q., Pan J., Yang J. (2011). Genetic variation in wild and cultured populations of the freshwater prawn, *Macrobrachium nipponense*, in China. J. World Aquac. Soc..

[15] Li X., Liu R., Liang X., Chen G. (2007). Fauna Sinica: Invertebrata. Crustacea, Decapoda, Palaemonoidea. Fauna Sinica: Invertebrata.

[16] Sambrook J., Russell D. (2001). Molecular Cloning: A Laboratory Manual.

[17] Jiang B., Fu J., Dong Z., Fang M., Zhu W., Wang L. (2019). Maternal ancestry analyses of red tilapia strains based on D-loop sequences of seven tilapia populations. PeerJ.

[18] Hall T.A. (1999). BioEdit: A user-friendly biological sequence alignment editor and analysis program for Windows 95/98/NT. Nucleic Acids Symposium Series.

[19] Excoffier L., Lischer H.E.L. (2010). Arlequin suite ver 3.5: A new series of programs to perform population genetics analyses under Linux and Windows. Mol. Ecol. Resour..

[20] Kimura M. (1980). A simple method for estimating evolutionary rates of base substitutions through comparative studies of nucleotide sequences. J. Mol. Evol..

[21] Benjamini Y., Hochberg Y. (1995). Controlling the false discovery rate: A practical and powerful approach to multiple testing. J. R. Stat. Soc. Ser. B-Stat. Methodol..

[22] Leigh J.W., Bryant D. (2015). POPART: Full-feature software for haplotype network construction. Methods Ecol. Evol..

[23] Kumar S., Stecher G., Tamura K. (2016). MEGA7: Molecular evolutionary genetics analysis version 7.0 for bigger datasets. Mol. Biol. Evol..

[24] Ou H.D., Cai S.R., Fan W., Qiu J.L., Mu X.L., Zhou T., Yang X.K., Picco L. (2024). Sustaining the Pearl River: A Critical Review of Changes in Fluvial Geomorphological Processes and the Driving Forces in the Pearl River Basin. Water.

[25] Oget C., Servin B., Palhiere I. (2019). Genetic diversity analysis of French goat populations reveals selective sweeps involved in their differentiation. Anim. Genet..

[26] Ujvari B., Klaassen M., Raven N., Russell T., Vittecoq M., Hamede R., Thomas F., Madsen T. (2018). Genetic diversity, inbreeding and cancer. Proc. R. Soc. B-Biol. Sci..

[27] Zhang D.-Y., Liu X.-M., Huang W.-J., Wang Y., Anwarullah K., Luo L.-F., Gao Z.-X. (2023). Whole-genome resequencing reveals genetic diversity and signatures of selection in mono-female grass carp (*Ctenopharyngodon idella*). Aquaculture.

[28] Lin M.H., Liang X.F., Lu K., Zeng M., Gao J.J., Dou Y.Q., Kuang Y.L., Zhang Q.W. (2025). Genetic Diversity in Three Sinipercine Fishes Based on Mitochondrial D-Loop and COX1 Sequences. Fishes.

[29] Iko R., Gao Z.J., Jiang S.F., Xiong Y.W., Zhang W.Y., Qiao H., Jin S.B., Fu H.T. (2025). Genetic Diversity and Population Structure of *Macrobrachium nipponense* Populations in the Saline-Alkaline Regions of China. Animals.

[30] Zhao L.J., Zhou X.Y., Liu Q.G., Zhang H. (2013). Genetic variation and Phylogeography of *Sinibrama macrops* (Teleostei: Cyprinidae) in Qiantang River Basin, China. Biochem. Syst. Ecol..

[31] Zhao L.J., Chenoweth E.L., Liu Q.G. (2018). Population structure and genetic diversity of Sinibrama macrops from Ou River and Ling River based on mtDNA D-loop region analysis, China. Mitochondrial DNA Part A.

[32] Wang Y.J., Lu J.H., Liu Z., Zhang J.P. (2021). Genetic diversity *of Gymnocypris chilianensis* (Cypriniformes, Cyprinidae) unveiled by the mitochondrial DNA D-loop region. Mitochondrial DNA Part B-Resour..

[33] Yuan X., Yang X., Ge H., Li H. (2019). Genetic Structure of *Spinibarbus caldwelli* Based on mtDNA D-Loop. Agric. Sci..

[34] Sun P., Yu J.Y., Tang B.J., Liu Z.D. (2021). Gene variation and population structure of *Pampus chinensis* in the China coast revealed by mitochondrial control region sequences. Mitochondrial DNA Part B-Resour..

[35] Fang D.A., Luo H., He M., Mao C.C., Kuang Z., Qi H.F., Xu D.P., Tan L.F., Li Y.D. (2022). Genetic Diversity and Population Differentiation of Naked Carp (*Gymnocypris przewalskii*) Revealed by Cytochrome Oxidase Subunit I and D-Loop. Front. Ecol. Evol..

