# Supplementary material for: Genetic Diversity and Population Structure of Wild Macrobrachium nipponense in the Pearl River Basin: A Treasury of Germplasm for Aquaculture and Conservation
Source: Animals (Basel). 2026 Sep 11;16(18):2869. doi: 10.3390/ani16182869 (PMC13603321; doi:10.3390/ani16182869)
Supplement: Supplementary file 1 [file animals-16-02869-s001.zip › animals-4518745-supplementary.pdf]

Table S1. Haplotype frequencies across 14 *Macrobrachium nipponense* populations

Haplotype	DH	DL	HP	HZ	XL	LL	NN	GP	JM	GZ	TX	ZQ	QZ	TE	Total
Hap_1:	3	2	1	0	1	0	0	1	0	0	1	0	0	4	13
Hap_2:	10	5	1	1	2	7	2	0	2	0	2	0	7	10	49
Hap_3:	2	3	3	0	0	0	4	0	0	0	0	0	0	0	12
Hap_4:	7	6	0	4	0	7	4	0	1	0	2	0	3	12	46
Hap_5:	2	2	0	0	1	3	0	0	0	0	0	0	0	0	8
Hap_6:	3	3	0	0	2	7	0	0	0	0	0	0	0	1	16
Hap_7:	1	1	0	1	0	1	2	0	0	0	0	0	0	0	6
Hap_8:	1	3	0	13	0	0	9	6	12	0	11	0	0	2	57
Hap_9:	1	0	0	1	12	0	0	0	0	0	0	0	0	0	14
Hap_10:	0	1	0	0	0	0	0	0	0	0	0	0	0	0	1
Hap_11:	0	1	0	0	0	2	1	0	0	0	0	0	0	0	4
Hap_12:	0	1	0	0	0	0	0	0	0	0	0	0	0	0	1
Hap_13:	0	1	0	0	0	1	0	0	0	0	0	0	0	0	2
Hap_14:	0	1	0	0	0	0	0	0	0	0	0	0	0	0	1
Hap_15:	0	0	17	0	0	0	0	0	0	0	0	0	3	0	20
Hap_16:	0	0	1	0	0	0	0	0	0	0	0	0	0	0	1
Hap_17:	0	0	1	0	0	0	0	0	0	0	0	0	0	0	1
Hap_18:	0	0	2	0	0	0	0	0	0	0	0	0	0	0	2
Hap_19:	0	0	1	0	0	0	0	0	0	0	0	0	0	0	1
Hap_20:	0	0	1	0	0	0	0	0	0	0	0	0	0	0	1
Hap_21:	0	0	1	0	0	0	0	0	0	0	0	0	0	0	1
Hap_22:	0	0	1	0	0	0	0	0	0	0	0	0	0	0	1
Hap_23:	0	0	0	1	5	1	0	0	0	0	1	0	0	1	9
Hap_24:	0	0	0	1	0	0	0	0	0	0	0	0	0	0	1
Hap_25:	0	0	0	1	3	1	0	2	0	0	0	0	0	0	7
Hap_26:	0	0	0	1	0	0	0	0	0	0	0	0	0	0	1
Hap_27:	0	0	0	1	0	0	0	0	0	0	0	0	0	0	1
Hap_28:	0	0	0	1	0	0	0	0	0	0	0	0	0	0	1
Hap_29:	0	0	0	1	0	0	0	0	0	0	0	0	0	0	1
Hap_30:	0	0	0	1	0	0	0	0	1	0	0	0	0	0	2
Hap_31:	0	0	0	1	0	0	0	0	0	0	0	0	0	0	1
Hap_32:	0	0	0	1	0	0	0	0	0	0	0	0	0	0	1
Hap_33:	0	0	0	0	2	0	0	0	0	0	0	0	0	0	2
Hap_34:	0	0	0	0	2	0	0	0	0	0	0	0	0	0	2
Hap_35:	0	0	0	0	0	0	1	0	0	0	0	0	0	0	1
Hap_36:	0	0	0	0	0	0	2	0	0	0	0	0	0	0	2
Hap_37:	0	0	0	0	0	0	1	0	0	0	0	0	0	0	1
Hap_38:	0	0	0	0	0	0	1	0	0	0	0	0	0	0	1
Hap_39:	0	0	0	0	0	0	1	0	0	0	0	0	0	0	1
Hap_40:	0	0	0	0	0	0	1	0	0	0	0	0	0	0	1
Hap_41:	0	0	0	0	0	0	1	0	0	0	0	0	0	0	1
Hap_42:	0	0	0	0	0	0	0	2	0	0	0	0	0	0	2
Hap_43:	0	0	0	0	0	0	0	2	0	0	0	0	0	0	2
Hap_44:	0	0	0	0	0	0	0	1	0	0	0	0	0	0	1
Hap_45:	0	0	0	0	0	0	0	1	0	0	0	0	0	0	1
Hap_46:	0	0	0	0	0	0	0	1	0	0	0	0	0	0	1
Hap_47:	0	0	0	0	0	0	0	1	0	0	0	0	0	0	1
Hap_48:	0	0	0	0	0	0	0	1	0	0	0	0	0	0	1

Haplotype	DH	DL	HP	HZ	XL	LL	NN	GP	JM	GZ	TX	ZQ	QZ	TE	Total
Hap_49：	0	0	0	0	0	0	0	1	0	0	0	0	0	0	1
Hap_50：	0	0	0	0	0	0	0	2	0	0	0	0	0	0	2
Hap_51：	0	0	0	0	0	0	0	1	0	0	0	0	0	0	1
Hap_52：	0	0	0	0	0	0	0	1	0	0	0	0	0	0	1
Hap_53：	0	0	0	0	0	0	0	2	0	0	0	0	0	0	2
Hap_54：	0	0	0	0	0	0	0	1	0	0	0	0	0	0	1
Hap_55：	0	0	0	0	0	0	0	1	0	0	0	0	0	0	1
Hap_56：	0	0	0	0	0	0	0	1	1	0	0	0	0	0	2
Hap_57：	0	0	0	0	0	0	0	2	0	0	1	0	0	0	3
Hap_58：	0	0	0	0	0	0	0	0	1	0	0	0	0	0	1
Hap_59：	0	0	0	0	0	0	0	0	2	0	0	0	0	0	2
Hap_60：	0	0	0	0	0	0	0	0	1	0	0	0	0	0	1
Hap_61：	0	0	0	0	0	0	0	0	1	0	0	0	0	0	1
Hap_62：	0	0	0	0	0	0	0	0	1	0	0	0	0	0	1
Hap_63：	0	0	0	0	0	0	0	0	1	0	0	0	0	0	1
Hap_64：	0	0	0	0	0	0	0	0	1	0	0	0	0	0	1
Hap_65：	0	0	0	0	0	0	0	0	2	0	0	0	0	0	2
Hap_66：	0	0	0	0	0	0	0	0	1	0	1	0	0	0	2
Hap_67：	0	0	0	0	0	0	0	0	1	0	0	0	0	0	1
Hap_68：	0	0	0	0	0	0	0	0	1	0	0	0	0	0	1
Hap_69：	0	0	0	0	0	0	0	0	0	1	0	0	0	0	1
Hap_70：	0	0	0	0	0	0	0	0	0	1	0	0	0	0	1
Hap_71：	0	0	0	0	0	0	0	0	0	1	0	0	0	0	1
Hap_72：	0	0	0	0	0	0	0	0	0	1	0	0	0	0	1
Hap_73：	0	0	0	0	0	0	0	0	0	1	0	0	0	0	1
Hap_74：	0	0	0	0	0	0	0	0	0	1	0	0	0	0	1
Hap_75：	0	0	0	0	0	0	0	0	0	1	0	0	0	0	1
Hap_76：	0	0	0	0	0	0	0	0	0	1	0	0	0	0	1
Hap_77：	0	0	0	0	0	0	0	0	0	1	0	0	0	0	1
Hap_78：	0	0	0	0	0	0	0	0	0	1	0	0	0	0	1
Hap_79：	0	0	0	0	0	0	0	0	0	1	0	0	0	0	1
Hap_80：	0	0	0	0	0	0	0	0	0	1	0	0	0	0	1
Hap_81：	0	0	0	0	0	0	0	0	0	1	0	0	0	0	1
Hap_82：	0	0	0	0	0	0	0	0	0	1	0	0	0	0	1
Hap_83：	0	0	0	0	0	0	0	0	0	1	0	0	0	0	1
Hap_84：	0	0	0	0	0	0	0	0	0	1	0	0	0	0	1
Hap_85：	0	0	0	0	0	0	0	0	0	1	0	0	0	0	1
Hap_86：	0	0	0	0	0	0	0	0	0	1	0	0	0	0	1
Hap_87：	0	0	0	0	0	0	0	0	0	1	0	0	0	0	1
Hap_88：	0	0	0	0	0	0	0	0	0	1	0	0	0	0	1
Hap_89：	0	0	0	0	0	0	0	0	0	1	0	0	0	0	1
Hap_90：	0	0	0	0	0	0	0	0	0	1	0	0	0	0	1
Hap_91：	0	0	0	0	0	0	0	0	0	1	0	0	0	0	1
Hap_92：	0	0	0	0	0	0	0	0	0	1	0	0	0	0	1
Hap_93：	0	0	0	0	0	0	0	0	0	1	0	0	0	0	1
Hap_94：	0	0	0	0	0	0	0	0	0	1	0	0	0	0	1
Hap_95：	0	0	0	0	0	0	0	0	0	1	0	0	0	0	1
Hap_96：	0	0	0	0	0	0	0	0	0	1	0	0	0	0	1
Hap_97：	0	0	0	0	0	0	0	0	0	1	0	0	0	0	1
Hap_98：	0	0	0	0	0	0	0	0	0	1	0	0	0	0	1

Haplotype	DH	DL	HP	HZ	XL	LL	NN	GP	JM	GZ	TX	ZQ	QZ	TE	Total
Hap_99:	0	0	0	0	0	0	0	0	0	0	1	0	0	0	1
Hap_100:	0	0	0	0	0	0	0	0	0	0	1	0	0	0	1
Hap_101:	0	0	0	0	0	0	0	0	0	0	1	0	1	0	2
Hap_102:	0	0	0	0	0	0	0	0	0	0	1	0	0	0	1
Hap_103:	0	0	0	0	0	0	0	0	0	0	1	0	0	0	1
Hap_104:	0	0	0	0	0	0	0	0	0	0	2	0	0	0	2
Hap_105:	0	0	0	0	0	0	0	0	0	0	2	0	0	0	2
Hap_106:	0	0	0	0	0	0	0	0	0	0	2	0	0	0	2
Hap_107:	0	0	0	0	0	0	0	0	0	0	0	1	0	0	1
Hap_108:	0	0	0	0	0	0	0	0	0	0	0	1	0	0	1
Hap_109:	0	0	0	0	0	0	0	0	0	0	0	1	0	0	1
Hap_110:	0	0	0	0	0	0	0	0	0	0	0	1	0	0	1
Hap_111:	0	0	0	0	0	0	0	0	0	0	0	1	0	0	1
Hap_112:	0	0	0	0	0	0	0	0	0	0	0	1	0	0	1
Hap_113:	0	0	0	0	0	0	0	0	0	0	0	1	0	0	1
Hap_114:	0	0	0	0	0	0	0	0	0	0	0	1	0	0	1
Hap_115:	0	0	0	0	0	0	0	0	0	0	0	1	0	0	1
Hap_116:	0	0	0	0	0	0	0	0	0	0	0	1	0	0	1
Hap_117:	0	0	0	0	0	0	0	0	0	0	0	1	0	0	1
Hap_118:	0	0	0	0	0	0	0	0	0	0	0	1	0	0	1
Hap_119:	0	0	0	0	0	0	0	0	0	0	0	1	0	0	1
Hap_120:	0	0	0	0	0	0	0	0	0	0	0	1	0	0	1
Hap_121:	0	0	0	0	0	0	0	0	0	0	0	1	0	0	1
Hap_122:	0	0	0	0	0	0	0	0	0	0	0	1	0	0	1
Hap_123:	0	0	0	0	0	0	0	0	0	0	0	1	0	0	1
Hap_124:	0	0	0	0	0	0	0	0	0	0	0	1	0	0	1
Hap_125:	0	0	0	0	0	0	0	0	0	0	0	1	0	0	1
Hap_126:	0	0	0	0	0	0	0	0	0	0	0	1	0	0	1
Hap_127:	0	0	0	0	0	0	0	0	0	0	0	1	0	0	1
Hap_128:	0	0	0	0	0	0	0	0	0	0	0	1	0	0	1
Hap_129:	0	0	0	0	0	0	0	0	0	0	0	1	0	0	1
Hap_130:	0	0	0	0	0	0	0	0	0	0	0	1	0	0	1
Hap_131:	0	0	0	0	0	0	0	0	0	0	0	1	0	0	1
Hap_132:	0	0	0	0	0	0	0	0	0	0	0	1	0	0	1
Hap_133:	0	0	0	0	0	0	0	0	0	0	0	1	0	0	1
Hap_134:	0	0	0	0	0	0	0	0	0	0	0	1	0	0	1
Hap_135:	0	0	0	0	0	0	0	0	0	0	0	1	0	0	1
Hap_136:	0	0	0	0	0	0	0	0	0	0	0	1	0	0	1
Hap_137:	0	0	0	0	0	0	0	0	0	0	0	0	1	0	1
Hap_138:	0	0	0	0	0	0	0	0	0	0	0	0	1	0	1
Hap_139:	0	0	0	0	0	0	0	0	0	0	0	0	1	0	1
Hap_140:	0	0	0	0	0	0	0	0	0	0	0	0	2	0	2
Hap_141:	0	0	0	0	0	0	0	0	0	0	0	0	1	0	1
Hap_142:	0	0	0	0	0	0	0	0	0	0	0	0	1	0	1
Hap_143:	0	0	0	0	0	0	0	0	0	0	0	0	1	0	1
Hap_144:	0	0	0	0	0	0	0	0	0	0	0	0	1	0	1
Hap_145:	0	0	0	0	0	0	0	0	0	0	0	0	1	0	1
Hap_146:	0	0	0	0	0	0	0	0	0	0	0	0	1	0	1
Hap_147:	0	0	0	0	0	0	0	0	0	0	0	0	1	0	1
Hap_148:	0	0	0	0	0	0	0	0	0	0	0	0	1	0	1

Haplotype	DH	DL	HP	HZ	XL	LL	NN	GP	JM	GZ	TX	ZQ	QZ	TE	Total
Hap_149:	0	0	0	0	0	0	0	0	0	0	0	0	1	0	1
Hap_150:	0	0	0	0	0	0	0	0	0	0	0	0	1	0	1
Hap_151:	0	0	0	0	0	0	0	0	0	0	0	0	1	0	1
Total	30	30	30	30	30	30	30	30	30	30	30	30	30	30	420
